# Liquid biopsy in non-small cell lung cancer: a meta-analysis of state-of-the-art and future perspectives

**DOI:** 10.3389/fgene.2023.1254839

**Published:** 2023-12-05

**Authors:** Sara Franzi, Gabriele Seresini, Paolo Borella, Paola Rafaniello Raviele, Gianluca Bonitta, Giorgio Alberto Croci, Claudia Bareggi, Davide Tosi, Mario Nosotti, Silvia Tabano

**Affiliations:** ^1^ Thoracic Surgery and Lung Transplantation, Fondazione IRCCS Ca’ Granda Ospedale Maggiore Policlinico, Milan, Italy; ^2^ Laboratory of Medical Genetics, Fondazione IRCCS Ca’ Granda Ospedale Maggiore Policlinico, Milan, Italy; ^3^ Division of Pathology, Fondazione IRCCS Ca’ Granda Ospedale Maggiore Policlinico, Milan, Italy; ^4^ Department of Pathophysiology and Transplantation, University of Milan, Milan, Italy; ^5^ Medical Oncology Fondazione IRCCS Ca’ Granda Ospedale Maggiore Policlinico, Milan, Italy

**Keywords:** liquid biopsy, CtDNA, EGFR, molecular markers, non-small cell lung cancer diagnosis

## Abstract

**Introduction:** To date, tissue biopsy represents the gold standard for characterizing non-small-cell lung cancer (NSCLC), however, the complex architecture of the disease has introduced the need for new investigative approaches, such as liquid biopsy. Indeed, DNA analyzed in liquid biopsy is much more representative of tumour heterogeneity.

**Materials and methods:** We performed a meta-analysis of 17 selected papers, to attest to the diagnostic performance of liquid biopsy in identifying EGFR mutations in NSCLC.

**Results:** In the overall studies, we found a sensitivity of 0.59, specificity of 0.96 and diagnostic odds ratio of 24,69. Since we noticed a high heterogeneity among different papers, we also performed the meta-analysis in separate subsets of papers, divided by 1) stage of disease, 2) experimental design and 3) method of mutation detection. Liquid biopsy has the highest sensitivity/specificity in high-stage tumours, and prospective studies are more reliable than retrospective ones in terms of sensitivity and specificity, both NGS and PCR-based techniques can be used to detect tumour DNA in liquid biopsy.

**Discussion:** Overall, liquid biopsy has the potential to help the management of NSCLC, but at present the non-homogeneous literature data, lack of optimal detection methods, together with relatively high costs make its applicability in routine diagnostics still challenging.

## 1 Introduction

Lung cancer is the main cause of cancer-related death, particularly regarding the broader group of non-small-cell lung cancer (NSCLC), with its three histologic variants: squamous cell carcinoma (SCC), adenocarcinoma (ADC) and large-cell carcinoma (Pujol et al., 2022; https://www.cap.org/). ADC is the most common subtype (Bray et al., 2018), accounting for 50% of all lung cancer diagnoses and showing an increase in occurrence in the latter decades (Barta et al., 2019).

NSCLC is often asymptomatic in its early phases and thus many patients are diagnosed only at an advanced stage, resulting in a poor prognosis, with a limited survival rate (approximately 18% at 5 years) (Wu et al., 2019; Abbasian et al., 2022).

### 1.1 Predictive molecular markers of non-small cell lung cancer

The identification of actionable molecular markers has transformed the management of NSCLC. Indeed, the genotype-directed treatment has significantly improved the overall survival (OS) in selected patients harbouring targetable genomic aberrations. Currently, at least 69% of patients with advanced NSCLC and mutations in *EGFR* (Epidermal Growth Factor), *KRAS* G12C (Kirsten Rat Sarcoma), *BRAF* V600E (V-RAF murine sarcoma viral oncogene homolog B), *ERBB2* (also known as *HER2*, human epidermal growth factor receptor 2), *ALK* (anaplastic lymphoma kinase gene), *ROS1* (ROS proto-oncogene 1, receptor tyrosine kinase), *MET* exon14 skipping (mesenchymal-epithelial transition), *RET* (rearranged during transfection), and *NTRK* (neurotrophic receptor tyrosine kinase 1) could receive FDA-approved (Food and Drug Administration) target therapies (Tsao et al., 2016). This, finally, results in a response rate to target therapy of about 60%–80% compared with 20%–45% in the standard chemotherapy-treated population, with median progression-free survival rising from 5-6 to 9–34 months in targetable patients (Palmero et al., 2021).

### 1.2 *EGFR* mutational status and target therapy response

Among the most predictive alterations, are the mutations in epidermal growth factor receptor (*EGFR*). EGFR is a trans-membrane receptor identified as an NSCLC oncogenic driver. In normal cells, it is activated by the binding of epidermal growth factor, which triggers different pathways involved in cell cycle progression, growth and angiogenesis (Casula et al., 2023). Mutations in *EGFR* lead to its constitutive activation, protein over-expression, and tumour progression (Bethune et al., 2010). *EGFR* mutation status is currently investigated to characterize NSCLC patients and to guide pharmacological treatment. Indeed, specific *EGFR* mutations confer sensitivity to selective EGFR-TKI inhibitors, thus allowing a targeted therapy, based on the molecular profile of the tumour, with the potential of improving the patient’s overall and progression-free survival, compared to standard chemotherapy (Arbour and Riely, 2019; Cheema et al., 2020). In detail, in NSCLC, *EGFR* presents with recurrent hot-spot alterations (single nucleotide missense variant as well as small insertion/deletions) at exons 18 to 21, codifying for the tyrosine kinase domain. The highest proportion of gene alterations (80%–90%) is represented by deletions within exon 19 and the point mutation c.2573T>G, p.L858R, at exon 21. Notably, patients harbouring exon 19 deletions have a better outcome, compared to patients with p.L858R, when treated with TKIs (Lino et al., 2023). The remaining 10%–20% of pathogenic *EGFR* variants are defined as “uncommon mutations” (Attili et al., 2022). Finally, *EGFR* can also have mutations that confer resistance to TKI inhibitors (e.g., p.T790M). The resistance generally occurs as a consequence of the treatment with TKI inhibitors in patients who showed a previous sensitizing *EGFR* mutation (Wu and Shih, 2018).

### 1.3 Tissue biopsy vs. liquid biopsy: state-of-the-art

At present, tissue biopsy (TB) is considered the gold standard for tumour diagnosis and molecular investigation of predictive biomarkers. Nevertheless, it is invasive for patients and has several limitations, mainly related to intra-tumour heterogeneity (i.e., different regions of the same tumour can bring different molecular alterations), as well as inter-tumour heterogeneity (i.e., different molecular profiles between the primary tumour and local or distant metastases of the same patient) (Gerlinger et al., 2012), both making tissue biopsy unrepresentative of the complete genetic makeup of the neoplasia. In addition, tumours can dynamically change over time, with the emergence of treatment-resistant subclones not detectable in the biopsy of the primary tumour (Bedard et al., 2013). Moreover, limiting factors in biomarker testing from tissue biopsy include the adequate quality of nucleic acids (DNA and RNA quality sub-optimal in formalin-fixed tissue, which is the routinary source of tumour tissue in molecular pathology diagnostics) as well as the availability of a sufficient amount of tumour tissue (e.g., tumour cellularity and size of the specimen) due to small tissue-samples delivered per patient respect to the increasing number of molecular markers that need to be investigated.

To overcome these limitations, liquid biopsy (LB), consisting of the analysis of tumour-released nucleic acids circulating in body fluids, such as blood, could provide a non-invasive and well-tolerated approach for tumour investigation. It allows the detection of circulating tumour DNA (ctDNA) carrying molecular tumour markers, which are more representative of the entire tumour and enable to follow disease evolution and dynamic changes in the molecular profile (Pérez-Callejo et al., 2016; Rijavec E et al., 2020; Bonanno et al., 2022; Pesta et al., 2022). In addition, in advanced/metastatic NSCLC, liquid biopsy has the potential to drive target therapy, by monitoring the response to treatment and identifying the possible molecular mechanisms of therapy resistance. Recently, Gristina et al. reported on the clinical potential of cfDNA in monitoring outcomes of NSCLC following first-line treatment. cfDNA has shown to be a reliable marker in helping clinicians in the decision-making process. Indeed, dynamic changes in cfDNA correlated with response to therapy with TKI and IO-based therapies. (Gristina et al., 2022). Finally, recent data have shown a significant ability of liquid biopsy in detecting minimal residual disease in early-stage lung cancer, underlying the potential application of LB in the adjuvant setting, in early detection of recurrence, and also for screening (Nigro et al., 2023).

Despite the evident practical advantages of liquid over tissue biopsy, LB is not yet widely adopted in clinical practice (Esagian et al., 2020) and standardized methods of LB investigation are currently lacking. Current Guidelines of ESMO (European Society for Medical Oncology) indicate liquid biopsy as complementary or alternative to tissue for biomarker evaluation of treatment-naïve NSCLC and recommend ctDNA evaluation only when a significant diagnostic delay is expected in obtaining tumour tissue for genotyping, when invasive procedures may be risky or not-indicated, or when bone would be the only site that could be biopsied (Pascual et al., 2022).

In the present manuscript, we performed a systematic review with meta-analysis to assess the state-of-the-art diagnostic potential of liquid biopsy in revealing *EGFR* predictive mutations in NSCLC patients. When different *EGFR* mutations were distinct in the text, we focused on exon 19 deletions.

## 2 Materials and methods

### 2.1 Literature search strategy/study design

We queried PubMed database up to December 2022, with no data restrictions, using the following search strategy: ((“Liquid Biopsy” [Mesh])) OR (((“Biopsy” [Mesh])) AND ((((“Exome” [Mesh]) OR “DNA/blood” [Mesh]) OR “RNA/blood” [Mesh]) OR “Neoplastic Cells, Circulating” [Mesh]))) AND “Lung Neoplasms” [Mesh].

The ethical approval was not applicable, because we performed a meta-analysis of the literature without involving human subjects.

### 2.2 Inclusion and exclusion criteria

PubMed database was independently screened by two Authors for articles of interest, according to the inclusion and exclusion criteria listed below; a double cross-check was performed and, in case of discrepancies, a third, independent supervisor was asked to review the collection. Articles were selected, included, and excluded following preferred reporting items for systematic reviews and meta-analysis (PRISMA) guidelines (PRISMA (prisma-statement.org). The inclusion criteria were: 1) human-based studies; 2) studies including at least 20 patients; 3) the absolute number of true positive (TP), true negative (TN), false positive (FP) and false negative (FN) presented in a 2X2 contingency table or easily deducible from the results section. The exclusion criteria comprised: 1) animal studies; 2) not sufficient data to construct a 2X2 contingency table; 3) reviews, meta-analyses, comments, and case reports.

### 2.3 Statistical analysis

A 2X2 table was generated including the absolute number of TP, TN, FP and FN, coupled with sensitivity and specificity data, to assess the diagnostic power of liquid biopsy in comparison with tissue biopsy.

We performed the bivariate Reitsma model (Reitsma et al., 2005) to explore the correlation between the logit of True Positive Rate (TPR) and logit of False Positive Rate (FPR); the confidence interval for correlation was estimated by the semi-parametric bootstrap percentile method. Separate meta-analyses of TPR and FPR were performed using the random-effects frequentist meta-analysis. Sensitivity (SE) and Specificity (SP) were pooled by generalized linear mixed models (GLMM) with logit transformation (Lin and Chu, 2020) by using the maximum likelihood to estimate the between-study variance. Clopper–Pearson confidence intervals were computed for an individual study. Diagnostic odd ratio (DOR), positive (PLR) and negative (NLR) likelihood ratios were pooled using the inverse-variance weighted random-effects frequentist meta-analysis with DerSimonian–Laird estimator for between-study variance (DerSimonian and Laird, 1986). Statistical heterogeneity was evaluated by the I^2^ index: a value ≤25% was defined as low heterogeneity, a value between 50% and 75% as moderate heterogeneity, and 75% or larger as high heterogeneity (Higgins and Thompson, 2002).

The 95% confidence intervals (95% CI) for pooled effect estimates were based on standard normal quantile. The prediction interval for the treatment effect of a new study was calculated according to Borenstein et al. (Borenstein et al., 2009). The one-leave-out sensitivity analysis was also performed. The continuity correction of 0.5 in studies with zero cell frequencies was used. The estimation of projected predictive values was based on a prevalence range and pooled (meta-analytical) sensitivities and specificities. All the confidence intervals were computed at a confidence level equal to 95%.

All the analyses and graphical representations were carried out using R version 3.2.2 software (R Core Team. 2023: https://www.R-project.org/with meta (Balduzzi et al., 2019) and mada packages (Doebler and Sousa-Pinto, 2022 mada: Meta-Analysis of Diagnostic Accuracy. R package version 0.5.11, https://CRAN.R-project.org/package=mada).

### 2.4 Subgroups stratification

Since we noticed a high variability in the results among different papers, mainly to the heterogeneity of the included studies, we also conducted statistical analyses by dividing patients into subgroups according to the following criteria: 1) tumour stage: high-stage (IIIB and IV) *versus* low-stage/locally advanced (I, II, IIIA); 2) experimental design: LB performed only in samples *EGFR*-positive at TB (retrospective studies) *versus* LB consecutively performed in both *EGFR*-positive and negative samples (prospective studies); 3) method of *EGFR* mutation detection: Next-Generation-Sequencing (NGS) *versus* PCR-based methods (see Table 1).

**TABLE 1:** Characteristics of the 17 studies included in the meta-analysis.

First author, year of publication	Country	Nr of patients	Investigated molecular marker	Tumour stage	Experimental design	Method of detection	TP	FP	TN	FN
Rachiglio et al. (2016)	Italy	44	*EGFR*	III, IV	P	NGS	17	2	20	5
He et al. (2017)	China	120	*EGFR*	III, IV	R	ddPCR	80	0	34	26
Yang et al. (2017)	China	107	*EGFR*	I-IV	P	CastPCR	23	3	64	17
Ito et al. (2018)	Japan	162	EGFR del exon 19^(*)^	I-IV	P	PNA-LNA PCR	3	0	148	11
			*EGFR L858R*				8	0	146	8
			*EGFR minor*				0	0	158	4
Guo et al. (2018)	China	56	*EGFR*	I-IV	P	NGS	3	2	27	26
Wan et al. (2018)	United States of America	284	*EGFR*	I-II	P	ARMS-PCR	21	14	122	127
Schrock et al. (2019)	United States of America	33	*EGFR*	III-IV	R	NGS	20	0	18	9
Li et al. (2019)	United States of America	110	*EGFR*	IV	P	NGS	18	0	86	6
Ding et al. (2019)	Australia	26	EGFR del exon 19^(*)^	IV	R	ddPCR	11	0	10	5
			*EGFR L858R*				11	0	10	5
			*EGFR S7681I*				4	0	5	1
			*EGFR L861Q*				3	0	3	0
Papadopoulou et al. (2019)	Greece	36	*EGFR*	IV	P	NGS	3	2	31	0
Ito et al. (2022)	Japan	100	*EGFR*	I-II	R	ddPCR	12	0	0	88
Qvick et al. (2021)	Sweden	52	*EGFR*	II, IV	R	AVENIO ctDNA Surveillance ki (NGS)t	4	0	45	3
Park et al. (2021)	South Korea	26	*EGFR, KRAS,* and others	IV	P	NGS	111	11	87	53
Batra et al. (2022)	India	184	EGFR del exon 19^(*)^	III, IV	P	Cobas	35	1	148	0
Lin et al. (2021)	United States of America	71	*EGFR, KRAS,* and others	II, III, IV	P	NGS	27	3	18	23
Satapathy et al. (2021)	India	60	*EGFR*	IIIB, IV	P	ddPCR	4	0	26	10
Prabhash et al. (2022)	India	240	*EGFR*	IV	P	NGS	54	16	145	25

^(*)^ In the studies where different *EGFR* mutations were investigated, we only considered deletion in exon 19 (del ex 19). *EGFR*: epidermal growth factor; *KRAS*: Kirsten rat sarcoma virus; P: prospective studies in which patients *EGFR* ^*+/−*^ at tissue biopsy were subjected to liquid biopsy; R: retrospective studies in which only patients *EGFR +* at tissue biopsy were subjected to liquid biopsy. NGS: Next-Generation Sequencing; ddPCR: digital droplet polymerase chain reaction; CAST-PCR: competitive allele-specific TaqMan PCR; PNA-LNA PCR: peptide nucleic acid-locked nucleic acid PCR; ARMS-PCR: amplification-refractory mutation system; TP: true positive (positive to tissue and liquid biopsy); FP false positive (positive to liquid biopsy and negative to tissue biopsy); TN: negative to tissue and liquid biopsy); FN: false negative (negative to liquid biopsy and positive to tissue positive).

## 3 Results

### 3.1 Search results

**FIGURE 1:**
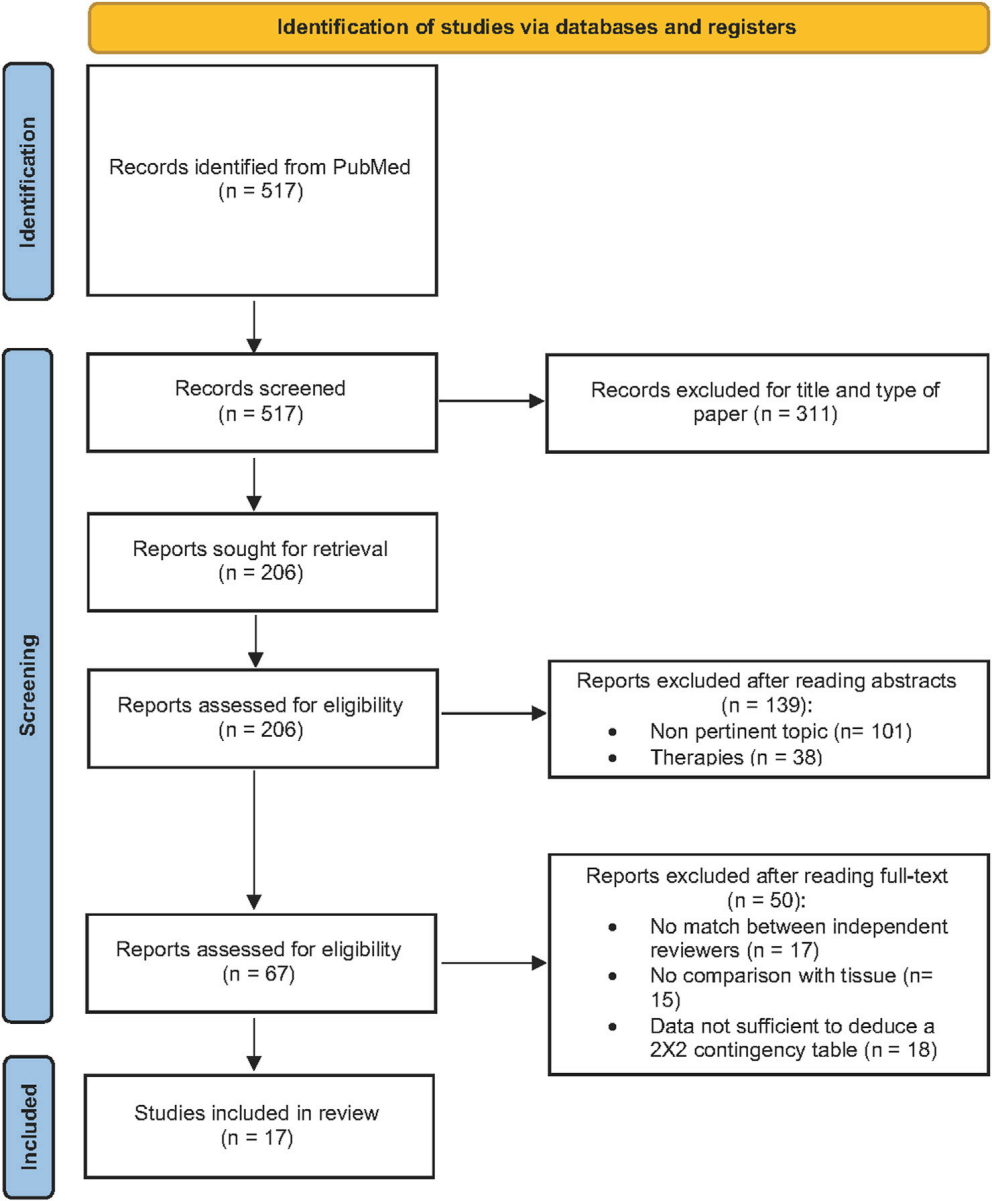
PRISMA flowchart of the literature selection process.

Literature search generated 517 papers: after reading the titles, abstracts and full-text, 17 articles were included in the meta-analysis (Rachiglio et al., 2016; He et al., 2017; Yang et al., 2017; Guo et al., 2018; Ito K et al., 2018; Wan et al., 2018; Ding et al., 2019; Li et al., 2019; Papadopoulou et al., 2019; Schrock et al., 2019; Lin et al., 2021; Park et al., 2021; Qvick et al., 2021; Satapathy et al., 2021; Batra et al., 2022; Ito M et al., 2022; Prabhash et al., 2022), summing up to a total of 1711 patients. Figure 1 represents the flowchart of the selection process, following PRISMA guidelines [PRISMA (prisma-statement.org)].

Table 1 Details of the characteristics of the 17 included studies: first author, year of publication, the country where the study was carried on, number of analyzed patients, and investigated molecular markers. We also indicated the tumour stage, the experimental design (P = prospective, R = retrospective, as specified in the Materials and Methods section) and the method of *EGFR* mutation investigation by liquid biopsy. Finally, we also reported the absolute numbers of True Positive (TP), False Positive (FP), True Negative (TN) and False Negative (FN) samples classified based on the *EGFR* mutation status of tissue biopsy: TP are samples positive at both TB and LB, TN are samples negative at both TB and LB, FP are samples positive in LB but negative in TB, FN are samples negative in LB but positive in TB.

### 3.2 Diagnostic power of overall liquid biopsy

**FIGURE 2:**
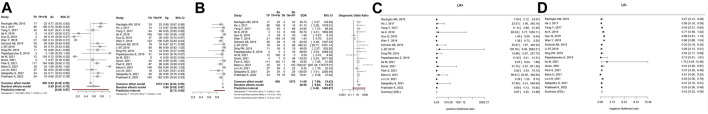
Forest plot of the diagnostic performance of liquid biopsy in overall studies, expressed with the following parameters: **(A)** Sensitivity (Se); **(B)** Specificity (Sp); **(C)** Diagnostic odds ratio (DOR); **(D)** positive and negative likelihood ratio (PLR and NLR). TP: true positive; FN: false negative; TN: true negative; FP: false positive.

Considering data from all 17 articles (1711 patients), LB sensitivity, specificity, diagnostic odds ratio (DOR), positive likelihood ratio (PLR), and negative likelihood ratio (NLR), were: 0.59 (95% CI: 0.41–0.75), 0.96 (95% CI: 0.92–0.97), 26.69 (95% CI: 9.62–74.07), 8.07 (95% CI: 4.35–14.98) and 0.43 (95% CI: 0.32–0.58), respectively (Figures 2A–D).

These overall results indicate that, though specificity was high and stable, sensitivity of LB was generally low and highly variable among studies, as shown by the heterogeneity index (I^2^ = 92%). This in turn negatively influenced Diagnostic Odd’s Ratio, which was also variable (I^2^ = 81%).

### 3.3 Diagnostic power of LB in high- and low-stage NSCLC

**FIGURE 3:**
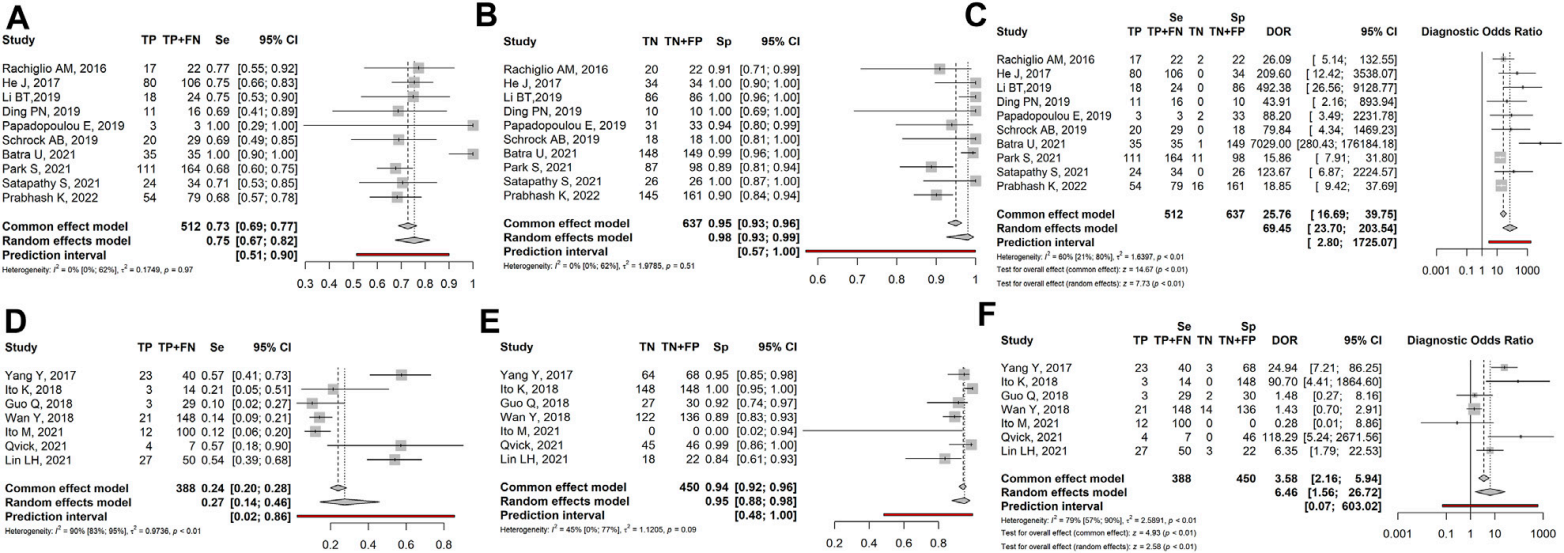
Forest plot of the diagnostic performance of liquid biopsy in the high-stage **(A–C)** vs. low-stage/locally advanced **(D–F)** NSCLC subgroups. **(A and D)** Sensitivity (Se); **(B and E)** Specificity (Sp); **(C and F)** Diagnostic odds ratio (DOR). TP: true positive; FN: false negative; TN: true negative; FP: false positive.

Since the results of studies including high-stage tumours could be different compared with lower-stage tumours because advanced tumours had a higher proportion of ctDNA, we separately analyzed high- (IIIB and IV) and low-stage/locally advanced (I, II, IIIA) tumours. Ten studies (1,115 patients) investigated high-stage tumours. Sensitivity, Specificity and DOR were 0.75 (95% CI: 0.67–0.82), 0.98 (95% CI: 0.93–0.99) and 69.45 (95% CI: 23.70–203.54), respectively, (Figures 3A–C). Seven studies (596 patients) investigated low-stage/locally advanced tumours. Sensitivity, Specificity and DOR were 0.27 (95% CI: 0.14–0.46), 0.95 (95% CI: 0.88–0.98) and 6.46 (95% CI: 1.56–26.72), respectively, (Figures 3D–F).

Overall, the sensitivity and diagnostic odds ratio were higher in the high-stage than in the low-stage tumours. While specificity maintained comparable values between the two groups (0.98 vs. 0.95 in high- and low-stage tumours respectively).

### 3.4 Diagnostics power of LB in prospective vs. retrospective studies

**FIGURE 4:**
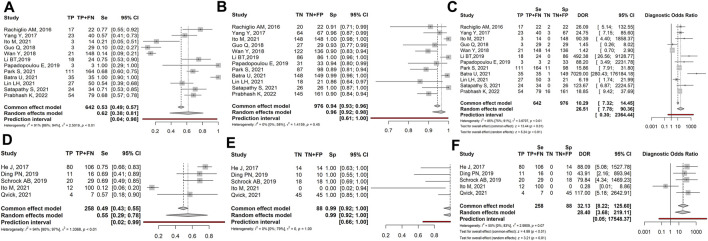
Forest plot of the diagnostic performance of liquid biopsy in prospective **(A–C)** vs. retrospective **(D–F)** NSCLC subgroups. **(A and D)** Sensitivity (Se); **(B and E)** Specificity (Sp); **(C and F)** Diagnostic odds ratio (DOR); TP: true positive; FN: false negative; TN: true negative; FP: false positive.

Based on the experimental design, papers could be divided into two groups: retrospective studies, in which LB was only performed in samples whose tissue biopsy resulted positive for *EGFR* mutations, and prospective studies, in which LB was performed in all samples, irrespective of *EGFR* mutational status at TB. Since this different inclusion criterion could influence the results, we separately examined the two groups. Twelve prospective studies (1,380 patients) showed sensitivity, specificity and DOR of: 0.62 (95% CI: 0.38–0.81), 0.96 (95% CI: 0.92–0.98) and 26.51 (95% CI: 7.78–90.36), respectively (Figures 4A–C). Five retrospective studies (331 patients) showed sensitivity, specificity and DOR of: 0.55 (95% CI: 0.29–0.78), 0.99 (95% CI: 0.92–1.00) and 28.40 (95% CI: 3.68–219.11), respectively (Figures 4D–F).

Overall, we observed that liquid biopsy showed slightly higher sensitivity in prospective than in retrospective studies (0.62 vs. 0.55). In contrast, specificity and diagnostic odds ratios were higher in retrospective than in prospective studies (0.99 and 28.40 vs. 0.96 and 26.51).

### 3.5 Diagnostic power of LB performed by NGS- vs. PCR-based investigation methods

Finally, we noticed that different techniques had been employed to analyze LB, and this could impact test performances: for this reason, we separately analyzed results obtained by NGS- and PCR-based methods.

**FIGURE 5:**
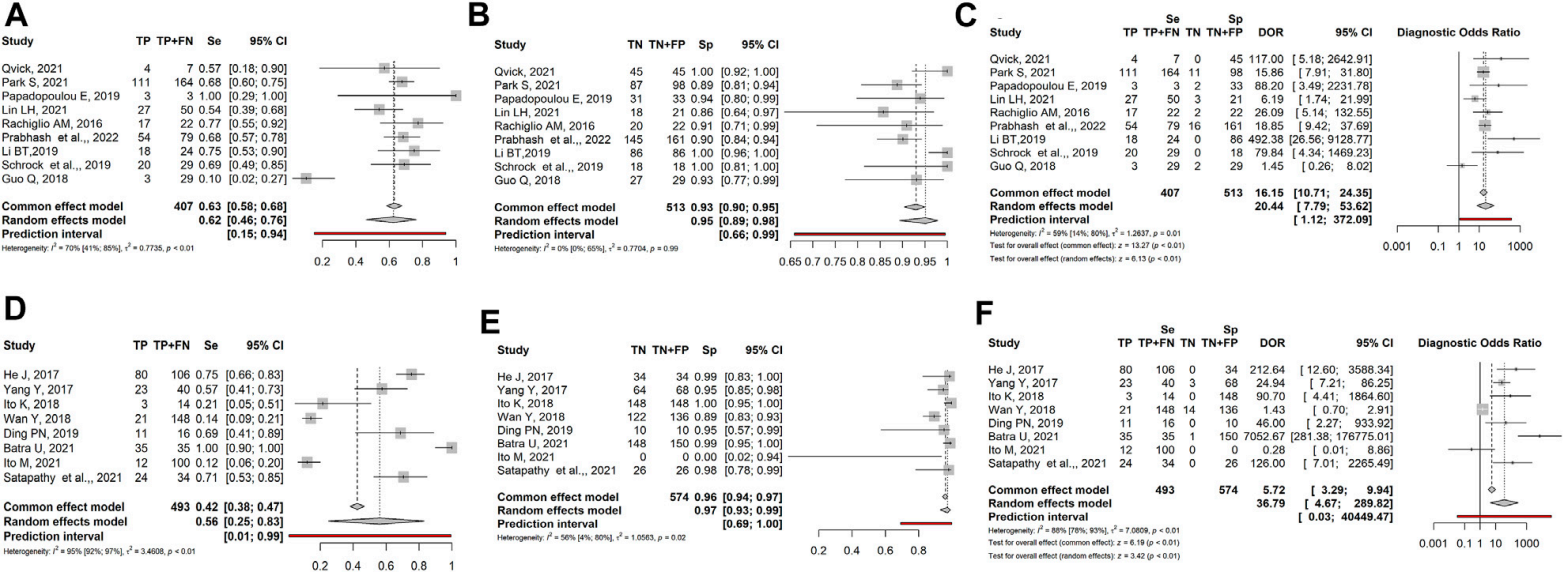
Forest plot of the diagnostic performance of liquid biopsy in NGS-based detection **(A–C)** vs. PCR-based **(D–F)** method NSCLC subgroups. **(A and )** Sensitivity (Se); **(B and E)** Specificity (Sp); **(C and F)** Diagnostic odds ratio (DOR); TP: true positive; FN: false negative; TN: true negative; FP: false positive.

Nine studies (668 patients) described samples analyzed by Next-Generation-Sequencing (NGS). Sensitivity, specificity, and DOR were 0.62 (95% CI: 0.46–0.76), 0.95 (95% CI: 0.89–0.98), and 20.44 (95% CI: 7.79–53.62), respectively (Figures 5A–C). Eight studies (1,043 patients) described samples analyzed by PCR-based methods, such as ddPPCR (digital droplet), CAST PCR (Competittive Allele-Specific TaqMan), PNA-LNA PCR (Peptide Nucleic Acid-Locked Nucleic Acid), ARMS PCR (Amplification Refractory Mutation System). Sensitivity, specificity, and DOR were 0.56 (95% CI: 0.25–0.83), 0.97 (95% CI: 0.93–0.99), and 36.79 (95% CI: 4.67–289.82), respectively (Figures 5D–F).

Though based on a small number of manuscripts, NGS showed slightly higher sensitivity than PCR-based techniques (0.62 vs. 0.56), while specificity was comparable (0.95 vs. 0.97). In contrast, PCR-based methods had a significantly higher diagnostic odds ratio than NGS (36.79 vs. 20.44).

## 4 Discussion

In the present meta-analysis, we evaluated data from 17 selected studies with a total of 1711 patients to investigate the diagnostic power of liquid biopsy in identifying *EGFR*-sensitizing mutations in NSCLC patients. Overall, the obtained results showed that LB has a sensitivity and specificity of 0.59 and 0.96, respectively. These values are in line with literature on NSCLC and other tumours (Zhu et al., 2020; Wang et al., 2021) and highlight an apparent low sensitivity of LB in detecting tumour mutations, while specificity appears high. However, when analyzing the manuscripts in detail, we noticed experimental variability among studies, especially regarding patients’ tumour stage (high-stage or low-stage), study design (prospective or retrospective studies), and methods of mutation detection (NGS- or PCR-based methods). These differences were able to modify the diagnostic performances of LB. For this reason, the overall results appeared highly heterogeneous, as indicated by I^2^ > 80% with the random model and by the confidence intervals, that were very wide for sensitivity, specificity and DOR (Figure 2). To overcome the heterogeneity of the results, and obtain more informative data, we separated the 17 articles into different subgroups, based on the above-mentioned variables, and made statistical analyses on grouped studies.

When dividing results based on the tumour stage, we observed in the high-stage subgroup the highest sensitivity (0.75) coupled with the lowest heterogeneity ( I^2^ = 0%), and the highest DOR (69.45 - Figures 3A–C). In detail, sensitivity ranged from 0.69 (Ding et al., 2019; Schrock et al., 2019) to 1.0 (Papadopoulou et al., 2019; Batra et al., 2022). Similarly, specificity showed high but variable values (from 0.89 to 1.0). As a consequence, DOR was also variable, ranging from 23.7 to 203.5. On the other hand, early-stage/locally advanced tumours showed the lowest sensitivity (0.27) and DOR (6.46), though specificity remained high (0.95–Figures 3D–F). Indeed, sensitivity was lower than 0.21 in 3 out of 7 cases, due to the high rate of FN samples and specificity was highly variable. Taken together, these findings indicate that the diagnostic performance of LB in NSCLC is influenced by the tumour stage and increases with the increase of tumour aggressiveness. It is conceivable that advanced-stage tumours, characterized by a high rate of apoptosis/necrosis, would release a higher amount of circulating DNA compared to early ones. Accordingly, literature data indicate that the ctDNA fraction varies based on tumour burden and stage, ranging from ≤0.01 to 0.1% in early-stage to ≥5–10% in advanced tumours (Vlataki et al., 2023).

Thus, LB could be properly used to follow the molecular evolution of the tumour over time and to monitor the response to treatment in advanced-stage tumours. Of note, even if in high-stage tumours LB sensitivity is high, it does not reach the same performance as TB. To increase LB sensitivity, blood samples could be repeated at different times or other biological fluids (e.g., saliva, urine, sputum) could be analysed as an alternative or in combination with plasma (Lino et al., 2023; Xin et al., 2023). On the other hand, LB does not seem to be indicated for early-stage tumours, showing a high rate of FN results, possibly due to the scarcity/lack of tDNA shed into circulation, or to the technical limits of current detection methods (Qiu et al., 2023).

The comparison between prospective and retrospective studies evidenced that the different experimental designs could generate discrepancies in the results, that need to be elucidated. In prospective cases, specificity had a mean value of 0.96, with values ranging from 0.86 (Lin et al., 2021) to 1.00 (Ito et al., 2018; Li et al., 2019; Sathapaty et al., 2021). Retrospective studies showed a specificity of 1.00 in all cases (except for Ito et al., 2022, in which the absence of TN cases resulted in a specificity of 0.00); however, specificity in the latter group was not realistic, since it was biased by the absence of false positive results. Sensitivity was higher in prospective than retrospective studies (0.62 vs. 0.55, respectively) probably due to the high proportion of FN in retrospective studies. Notably, both subsets showed high heterogeneity (I^2^ = 94% and 91%, respectively). Overall, these results seem indicate that prospective studies are more reliable than retrospective ones, in defining sensitivity and specificity in real-world diagnostic workflow.

Another factor affecting LB diagnostic performance could be the method of mutation detection. At present, NGS and PCR-based techniques are employed for ctDNA detection; however, ctDNA assessment is hampered by its low amount in the bloodstream, requiring the need for even more sensitive techniques for detection and quantification. Comparing NGS with PCR-based methods we found similar sensitivity and specificity values whereas DOR, though very heterogeneous, was higher in the PCR-based subgroup. Of note, in the PCR-based group, the use of different techniques affects the performance. Overall, none of the methods can be considered optimal, since each one of them shows “pros and cons”. NGS is widely used for the detection of ctDNA: it allows simultaneous sequencing of different genomic regions in many samples, it is also able to quantitate gene copy number variations, including gene amplification, and to identify chromosomal rearrangements such as oncogenic fusions. Besides, NGS is able to calculate the frequency of the variant allele (Rolfo et al., 2018; Fernandes et al., 2021; Pesta et al., 2022). NGS also allows the comprehension of tumour genetic features and provides crucial information on tumour microenvironment and immune response, that might drive the response to immuno-therapy. (Qiu et al., 2023). PCR-based techniques are characterized by a short turnaround time and easy interpretation of results, but allow the detection of only one alteration at a time. Of note, they are very sensitive in the detection of low-frequency alleles; particularly, ddPCR (<0.01 of mutated alleles) and COBAS have proved adequate in the genetic profiling of tumours (Rolfo et al., 2018; Vlataki et al., 2023).

According to current guidelines (Rolfo et al., 2018), both NGS and PCR-based methods can be used by LB: in the presence of *EGFR*-sensitizing mutations, patients can initiate target therapy. However, given the low sensitivity of LB compared to TB, negative results should be considered non-conclusive and be implemented by other TB tests (Rolfo et al., 2018). To overcome this limitation, recently, Kwon et al. reported on the importance of integrating multi-omics data into machine learning analyses to significantly improve accuracy in cancer diagnosis (Kwon et al., 2023).

## 5 Conclusion

In conclusion, though LB has the potential to help the management of NSCLC in advanced-stage patients, at present non-homogeneous literature data, lack of standardized detection methods, together with relatively high costs, make its applicability in routine diagnostics still challenging.

## Data Availability

The original contributions presented in the study are included in the article/supplementary material, further inquiries can be directed to the corresponding author.

## References

[B1] Abbasian M. H. Ardekani A. M. Sobhani N. Roudi R. (2022). The role of genomics and proteomics in lung cancer early detection and treatment. Cancers 14, 5144. 10.3390/cancers14205144 36291929 PMC9600051

[B2] Arbour K. C. Riely G. J. (2019). Systemic therapy for locally advanced and metastatic non-small cell lung cancer: a review. JAMA 322, 764–774. 10.1001/jama.2019.11058 31454018

[B3] Attili I. Passaro A. Pisapia P. Malapelle U. de Marinis F. (2022). Uncommon *EGFR* compound mutations in non-small cell lung cancer (NSCLC): a systematic review of available evidence. Curr. Oncol. 29, 255–266. 10.3390/curroncol29010024 35049698 PMC8774526

[B4] Balduzzi S. Rücker G. Schwarzer G. (2019). How to perform a meta-analysis with R: a practical tutorial. Evidence-Based Ment. Health 22, 153–160. 10.1136/ebmental-2019-300117 PMC10231495 31563865

[B5] Barta J. A. Powell C. A. Wisnivesky J. P. (2019). Global epidemiology of lung cancer. Ann. Glob. Health 85, 8. 10.5334/aogh.2419 30741509 PMC6724220

[B6] Batra U. Nathany S. Sharma M. Jain P. Dhanda S. Singh H. (2022). *EGFR* detection by liquid biopsy: ripe for clinical usage. Future Oncol. 18, 85–92. 10.2217/fon-2021-0620 34704813

[B7] Bedard P. L. Hansen A. R. Ratain M. J. Siu L. L. (2013). Tumour heterogeneity in the clinic. Nature 501, 355–364. 10.1038/nature12627 24048068 PMC5224525

[B8] Bethune G. Bethune D. Ridgway N. Xu Z. (2010). Epidermal growth factor receptor (EGFR) in lung cancer: an overview and update. J. Thorac. Dis. 2, 48–51. 22263017 PMC3256436

[B9] Bonanno L. Dal Maso A. Pavan A. Zulato E. Calvetti L. Pasello G. (2022). Liquid biopsy and non-small cell lung cancer: are we looking at the tip of the iceberg? Br. J. Cancer 127, 383–393. 10.1038/s41416-022-01777-8 35264788 PMC9345955

[B10] Borenstein M. Hedges L. V. Higgins J. P. T. Rothstein H. R. (2009). Introduction to meta-analysis. Chichester, UK: John Wiley & Sons.

[B11] Bray F. Ferlay J. Soerjomataram I. Siegel R. L. Torre L. A. Jemal A. (2018). Global cancer statistics 2018: GLOBOCAN estimates of incidence and mortality worldwide for 36 cancers in 185 countries. Can. J. Clin. 68, 394–424. 10.3322/caac.21492 30207593

[B12] Casula M. Pisano M. Paliogiannis P. Colombino M. Sini M. C. Zinellu A. (2023). Comparison between three different techniques for the detection of EGFR mutations in liquid biopsies of patients with advanced-stage lung adenocarcinoma. Int. J. Mol. Sci. 24, 6410. 10.3390/ijms24076410 37047382 PMC10094170

[B13] Cheema P. K. Gomes M. Banerji S. Joubert P. Leighl N. B. Melosky B. (2020). Consensus recommendations for optimizing biomarker testing to identify and treat advanced *EGFR*-mutated non-small-cell lung cancer. Curr. Oncol. 27 (6), 321–329. 10.3747/co.27.7297 33380864 PMC7755440

[B14] DerSimonian R. Laird N. (1986). Meta-analysis in clinical trials. Control Clin. Trials 7, 177–188. 10.1016/0197-2456(86)90046-2 3802833

[B15] Ding P. N. Becker T. M. Bray V. J. Chua W. Ma Y. F. Lynch D. (2019). The predictive and prognostic significance of liquid biopsy in advanced epidermal growth factor receptor-mutated non-small cell lung cancer: a prospective study. Lung Cancer 134, 187–193. 10.1016/j.lungcan.2019.06.021 31319980

[B16] Doebler P. Sousa-Pinto B. (2022). Mada: meta-analysis of diagnostic accuracy. R package version 0.5.11.

[B17] Esagian S. M. Grigoriadou G. I. Nikas I. P. Boikou V. Sadow P. M. Won J. K. (2020). Comparison of liquid-based to tissue-based biopsy analysis by targeted next-generation sequencing in advanced non-small cell lung cancer: a comprehensive systematic review. J. Cancer Res. Clin. Oncol. 146, 2051–2066. 10.1007/s00432-020-03267-x 32462295 PMC7456570

[B18] Fernandes M. G. O. Cruz-Martins N. Souto Moura C. Guimarães S. Pereira Reis J. Justino A. (2021). Clinical application of next-generation sequencing of plasma cell-free DNA for genotyping untreated advanced non-small cell lung cancer. Cancers (Basel) 13, 2707. 10.3390/cancers13112707 34070940 PMC8199488

[B19] Gerlinger M. Rowan A. J. Horswell S. Math M. Larkin J. Endesfelder D. (2012). Intratumor heterogeneity and branched evolution revealed by multiregion sequencing. N. Engl. J. Med. 366, 883–892. 10.1056/NEJMoa1113205 22397650 PMC4878653

[B20] Gristina V. Barraco N. La Mantia M. Castellana L. Insalaco L. Bono M. (2022). Clinical potential of circulating cell-free DNA (cfDNA) for longitudinally monitoring clinical outcomes in the first-line setting of non-small-cell lung cancer (NSCLC): a real-world prospective study. Cancers 14, 6013. 10.3390/cancers14236013 36497493 PMC9735435

[B21] Guo Q. Wang J. Xiao J. Wang L. Hu X. Yu W. (2018). Heterogeneous mutation pattern in tumor tissue and circulating tumor DNA warrants parallel NGS panel testing. Mol. Cancer 17, 131. 10.1186/s12943-018-0875-0 30153823 PMC6114875

[B22] He J. Tan W. Ma J. (2017). Circulating tumor cells and DNA for real-time *EGFR* detection and monitoring of non-small-cell lung cancer. Future Oncol. 13, 787–797. 10.2217/fon-2016-0427 28073294

[B23] Higgins J. P. Thompson S. G. (2002). Quantifying heterogeneity in a meta-analysis. Stat. Med. 21, 1539–1558. 10.1002/sim.1186 12111919

[B25] Ito K. Suzuki Y. Saiki H. Sakaguchi T. Hayashi K. Nishii Y. (2018). Utility of liquid biopsy by improved PNA-LNA PCR clamp method for detecting EGFR mutation at initial diagnosis of non-small-cell lung cancer: observational study of 190 consecutive cases in clinical practice. Clin. Lung Cancer 19, 181–190. 10.1016/j.cllc.2017.10.017 29174086

[B26] Ito M. Miyata Y. Hirano S. Irisuna F. Kushitani K. Kai Y. (2022). Sensitivity and optimal clinicopathological features for mutation-targeted liquid biopsy in pN0M0 EGFR-mutant lung adenocarcinoma. J. Cancer Res. Clin. Oncol. 148, 1419–1428. 10.1007/s00432-021-03721-4 34218331 PMC11800930

[B27] Kwon H. K. Park U. H. Goh C. J. Park D. Lim Y. G. Kise Lee I. (2023). Enhancing lung cancer classification through integration of liquid biopsy multi-omics data with machine learning techniques. Cancer (Basel) 15, 4556. 10.3390/cancers15184556 PMC10526503 37760525

[B28] Li B. T. Janku F. Jung B. Hou C. Madwani K. Alden R. (2019). Ultra-deep next-generation sequencing of plasma cell-free DNA in patients with advanced lung cancers: results from the Actionable Genome Consortium. Ann. Oncol. 30, 597–603. 10.1093/annonc/mdz046 30891595 PMC6503621

[B29] Lin L. Chu H. (2020). Meta-analysis of proportions using generalized linear mixed models. Epidemiology 31, 713–717. 10.1097/EDE.0000000000001232 32657954 PMC7398826

[B30] Lin L. H. Allison D. H. R. Feng Y. Jour G. Park K. Zhou F. (2021). Comparison of solid tissue sequencing and liquid biopsy accuracy in identification of clinically relevant gene mutations and rearrangements in lung adenocarcinomas. Mod. Pathol. 34, 2168–2174. 10.1038/s41379-021-00880-0 34362997

[B31] Lino C. Barrias S. Chaves R. Adega F. Fernandes J. R. Martins-Lopes P. (2023). Development of a QCM-based biosensor for the detection of non-small cell lung cancer biomarkers in liquid biopsies. Talanta 260, 124624. 10.1016/j.talanta.2023.124624 37187027

[B32] Nigro M. C. Marchese P. V. Deiana C. Casadio C. Galvani L. Di Federico A. (2023). Clinical utility and application of liquid biopsy genotyping in lung cancer: a comprehensive review. Lung Cancer (Auckl) 14, 11–25. 10.2147/LCTT.S388047 36762267 PMC9904307

[B34] Palmero R. Taus A. Viteri S. Majem M. Carcereny E. Garde-Noguera J. (2021). Biomarker discovery and outcomes for comprehensive cell-free circulating tumor DNA versus standard-of-care tissue testing in advanced non-small-cell lung cancer. JCO Precis. Oncol. 5, 93–102. 10.1200/PO.20.00241 34994593

[B35] Papadopoulou E. Tsoulos N. Tsantikidi K. Metaxa-Mariatou V. Stamou P. E. Kladi-Skandali A. (2019). Clinical feasibility of NGS liquid biopsy analysis in NSCLC patients. PLoS One 14, e0226853. 10.1371/journal.pone.0226853 31860648 PMC6924668

[B36] Park S. Olsen S. Ku B. M. Lee M. S. Jung H. A. Sun J. M. (2021). High concordance of actionable genomic alterations identified between circulating tumor DNA-based and tissue-based next-generation sequencing testing in advanced non-small cell lung cancer: the Korean Lung Liquid versus Invasive Biopsy Program. Cancer 127, 3019–3028. 10.1002/cncr.33571 33826761

[B37] Pascual J. Attard G. Bidard F. C. Curigliano G. De Mattos-Arruda L. Diehn M. (2022). ESMO recommendations on the use of circulating tumour DNA assays for patients with cancer: a report from the ESMO Precision Medicine Working Group. Ann. Oncol. 33, 750–768. 10.1016/j.annonc.2022.05.520 35809752

[B38] Pérez-Callejo D. Romero A. Provencio M. Torrente M. (2016). Liquid biopsy-based biomarkers in non-small cell lung cancer for diagnosis and treatment monitoring. Transl. Lung Cancer Res. 5, 455–465. 10.21037/tlcr.2016.10.07 27826527 PMC5099509

[B39] Pesta M. Shetti D. Kulda V. Knizkova T. Houfkova K. Bagheri M. S. (2022). Applications of liquid biopsies in non-small-cell lung cancer. Diagnostics 12, 1799. 10.3390/diagnostics12081799 35892510 PMC9330570

[B40] Prabhash K. Biswas B. Khurana S. Batra U. Biswas G. Advani S. H. (2022). CONCORDANCE: a real-world evidence study to evaluate the concordance of detecting epidermal growth factor receptor (EGFR) mutation by circulating tumor DNA* versus tissue biopsy in patients with metastatic non-small cell lung cancer. Indian J. Cancer 59, S11–S18. 10.4103/ijc.ijc_438_21 35343188

[B41] Pujol N. Heeke S. Bontoux C. Boutros J. Ilié M. Hofman V. (2022). Molecular profiling in non-squamous non-small cell lung carcinoma: towards a switch to next-generation sequencing reflex testing. J. Pers. Med. 12, 1684. 10.3390/jpm12101684 36294823 PMC9605324

[B50] Qiu T. Zhi X. Ren S. (2023). Recent advance of next-generation sequencing in patients with lung cancer. Expert Rev. Mol. Diagn. 23, 959–970. 10.1080/14737159.2023.2260755 37750512

[B42] Qvick A. Stenmark B. Carlsson J. Isaksson J. Karlsson C. Helenius G. (2021). Liquid biopsy as an option for predictive testing and prognosis in patients with lung cancer. Mol. Med. 27, 68. 10.1186/s10020-021-00331-1 34217228 PMC8254966

[B43] Rachiglio A. M. Abate R. E. Sacco A. Pasquale R. Fenizia F. Lambiase M. (2016). Limits and potential of targeted sequencing analysis of liquid biopsy in patients with lung and colon carcinoma. Oncotarget 7, 66595–66605. 10.18632/oncotarget.10704 27448974 PMC5341823

[B44] R Core Team (2023). R: a language and environment for statistical computing. Vienna, Austria: R foundation for statistical computing.

[B45] Reitsma J. Glas A. Rutjes A. Scholten R. Bossuyt P. Zwinderman A. A. (2005). Bivariate analysis of sensitivity and specificity produces informative summary measures in diagnostic reviews. J. Clin. Epidemiol. 58, 982–990. 10.1016/j.jclinepi.2005.02.022 16168343

[B46] Rijavec E. Coco S. Genova C. Rossi G. Longo L. Grossi F. (2020). Liquid biopsy in non-small cell lung cancer: highlights and challenges. Cancers 12, 17. 10.3390/cancers12010017 PMC7017364 31861557

[B47] Rolfo C. Mack P. C. Scagliotti G. V. Baas P. Barlesi F. Bivona T. G. (2018). Liquid biopsy for advanced non-small cell lung cancer (NSCLC): a statement paper from the iaslc. J. Thorac. Oncol. 13, 1248–1268. 10.1016/j.jtho.2018.05.030 29885479

[B48] Satapathy S. Singh V. Nambirajan A. Malik P. S. Tanwar P. Mehta A. (2021). EGFR mutation testing on plasma and urine samples: a pilot study evaluating the value of liquid biopsy in lung cancer diagnosis and management. Curr. Probl. Cancer 45, 100722. 10.1016/j.currproblcancer.2021.100722 33712318

[B49] Schrock A. B. Welsh A. Chung J. H. Pavlick D. Bernicker E. H. Creelan B. (2019). Hybrid capture-based genomic profiling of circulating tumor DNA from patients with advanced non-small cell lung cancer. J. Thorac. Oncol. 14, 255–264. 10.1016/j.jtho.2018.10.008 30368012

[B51] Tsao A. S. Scagliotti G. V. Bunn P. A. Jr. Carbone D. P. Warren G. W. Bai C. (2016). Scientific advances in lung cancer. J. Thorac. Oncol. 11, 613–638. 10.1016/j.jtho.2016.03.012 27013409

[B52] Vlataki K. Antonouli S. Kalyvioti C. Lampri E. Kamina S. Mauri D. (2023). Circulating tumor DNA in the management of early-stage breast cancer. Cells 12, 1573. 10.3390/cells12121573 37371043 PMC10296563

[B53] Wan Y. Liu B. Lei H. Zhang B. Wang Y. Huang H. (2018). Nanoscale extracellular vesicle-derived DNA is superior to circulating cell-free DNA for mutation detection in early-stage non-small-cell lung cancer. Ann. Oncol. 29, 2379–2383. 10.1093/annonc/mdy458 30339193 PMC6311950

[B54] Wang N. Zhang X. Wang F. Zhang M. Sun B. Yin W. (2021). The diagnostic accuracy of liquid biopsy in EGFR-mutated NSCLC: a systematic review and meta-analysis of 40 studies. SLAS Technol. 26, 42–54. 10.1177/2472630320939565 32659150

[B55] Wu S. G. Shih J. Y. (2018). Management of acquired resistance to EGFR TKI-targeted therapy in advanced non-small cell lung cancer. Mol. Cancer 17, 38. 10.1186/s12943-018-0777-1 29455650 PMC5817870

[B56] Wu Z. Yang Z. Dai Y. Zhu Q. Chen L. A. (2019). Update on liquid biopsy in clinical management of non-small cell lung cancer. OncoTargets Ther. 12, 5097–5109. 10.2147/OTT.S203070 PMC6611714 31303765

[B57] Xin L. Yue Y. Zihan R. Youbin C. Tianyu L. Rui W. (2023). Clinical application of liquid biopsy based on circulating tumor DNA in non-small cell lung cancer. Front. Physiol. 14, 1200124. 10.3389/fphys.2023.1200124 37351260 PMC10282751

[B58] Yang Y. Shen X. Li R. Shen J. Zhang H. Yu L. (2017). The detection and significance of EGFR and BRAF in cell-free DNA of peripheral blood in NSCLC. Oncotarget 8, 49773–49782. 10.18632/oncotarget.17937 28572536 PMC5564806

[B59] Zhu Y. Zhang H. Chen N. Hao J. Jin H. Ma X. (2020). Diagnostic value of various liquid biopsy methods for pancreatic cancer: a systematic review and meta-analysis. Med. Baltim. 99, e18581. 10.1097/MD.0000000000018581 PMC7220382 32011436

